# Interleukin 15: A new intermediary in the effects of exercise and training on skeletal muscle and bone function

**DOI:** 10.1111/jcmm.70136

**Published:** 2024-11-27

**Authors:** Ziqiang Duan, Yang Yang, Mianhong Qin, Xuejie Yi

**Affiliations:** ^1^ School of Sports Health Shenyang Sport University Shenyang China; ^2^ School of Kinesiology Shanghai University of Sport Shanghai China; ^3^ Social Science Research Center Shenyang Sport University Shenyang China

**Keywords:** bone, exercise, interleukin‐15, locomotor system, metabolism, muscle, training

## Abstract

Interleukin‐15 (IL‐15), a pro‐inflammatory cytokine, is produced mainly by skeletal muscle cells, macrophages and epithelial cells. Recent research has demonstrated that IL‐15 is closely related to the functions of bone and skeletal muscle in the locomotor system. There is growing evidence that exercise, an important means to regulate the immune and locomotor systems, influences IL‐15 content in various tissues, thereby indirectly affecting the function of bones and muscles. Furthermore, the form, intensity, and duration of exercise determine the degree of change in IL‐15 and downstream effects. This paper reviews the structure, synthesis and secretion of IL‐15, the role of IL‐15 in regulating the metabolism of bone tissue cells and myofibers through binding to the IL‐15 receptor‐α (IL‐15Rα), and the response of IL‐15 to different types of exercise. This review provides a reference for further analyses of the role and mechanism of action of IL‐15 in the regulation of metabolism during exercise.

## 1 INTRODUCTION

**FIGURE 1:**
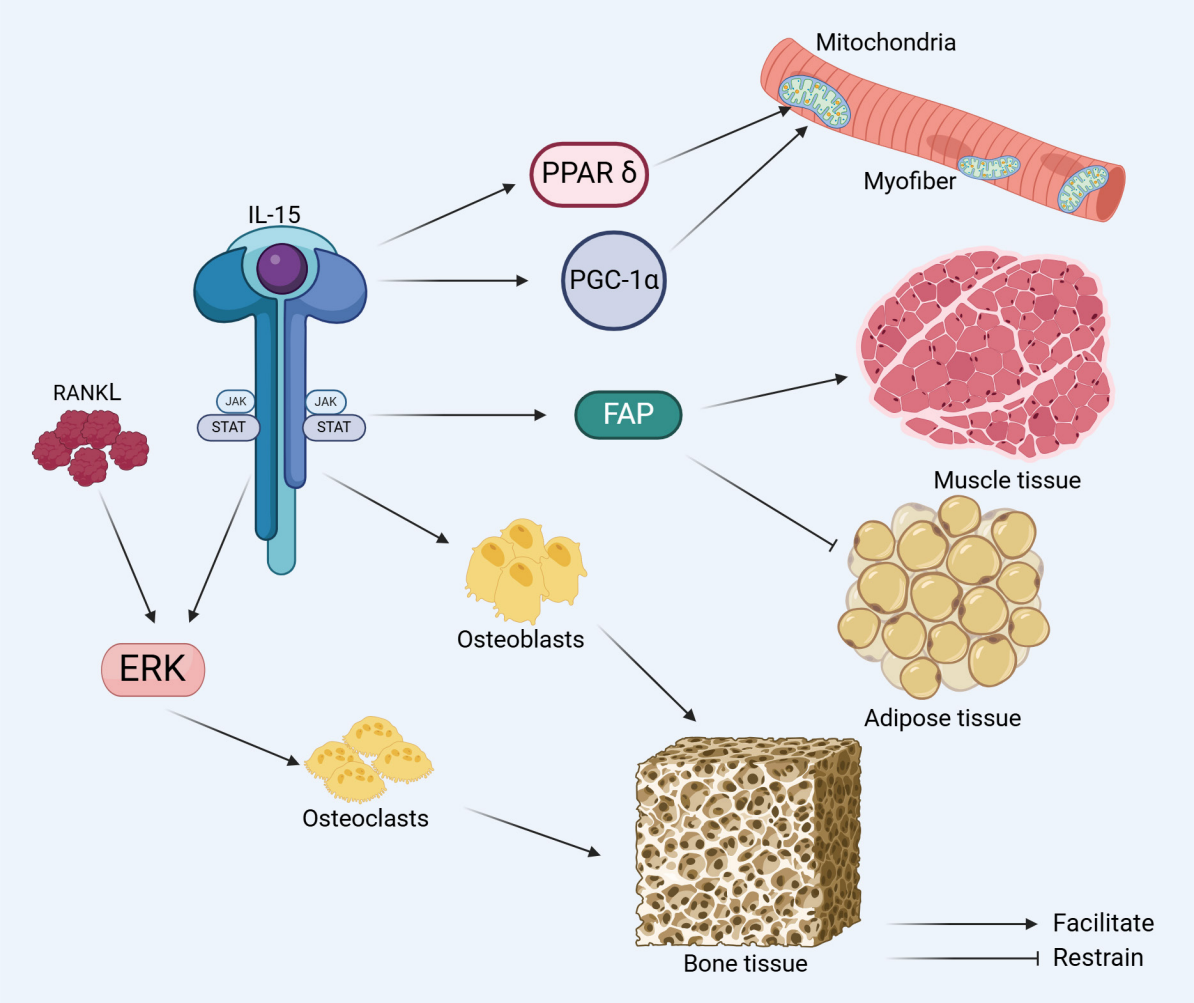
Role of IL‐15 in muscle and bone. (1) IL‐15 enhances mitochondrial biogenesis in myofibers and the skeletal muscle energy supply by promoting the expression of PPAR δ and PGC‐1α. (2) IL‐15 acts through the JAK/STAT pathway, inhibits the lipogenic differentiation of FAPs, and promotes myofiber regeneration. (3) IL‐15/IL‐15Rα signalling regulates the differentiation of osteoblasts and mediates the generation of osteocytes, thereby affecting the structure of bone tissue. (4) IL‐15 and RANKL act synergistically through the ERK signalling pathway to promote osteoclastogenesis and affect bone tissue structure.

**FIGURE 2:**
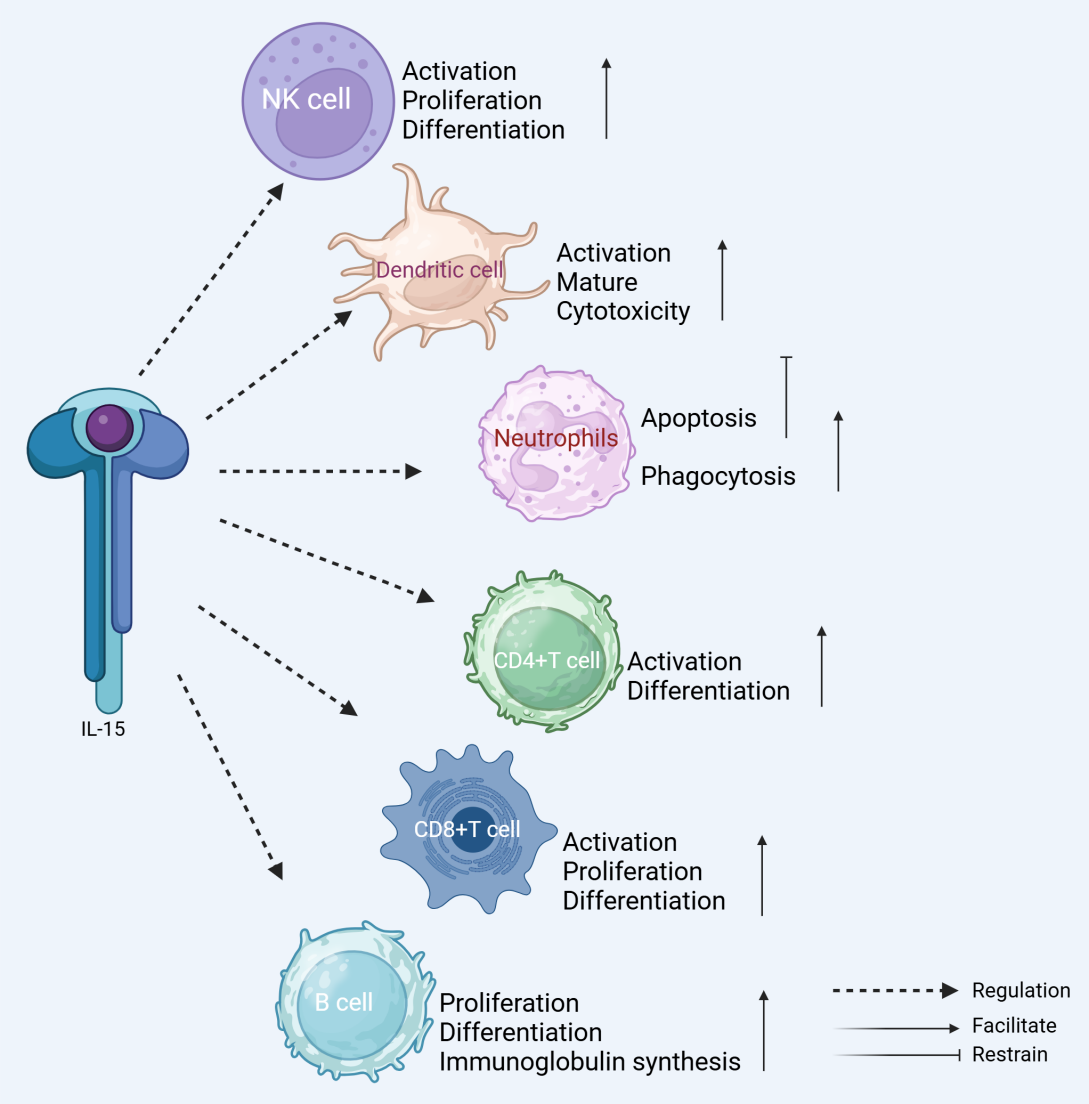
Significant effects of IL‐15 on immune cells. Effects of IL‐15 regulation on innate (NK cells, dendritic cells, and neutrophils) and adaptive (T and B cells) immune system.

Interleukin‐15 (IL‐15), discovered in 1994, is a pro‐inflammatory cytokine with functions in various immune cells.^1^ It is expressed in the locomotor system (skeletal muscle and bone) and, similar to other muscle‐secreted factors, exhibits pleiotropic effects.^2^ IL‐15 continuously and dynamically regulates bone metabolism and has important roles in osteogenesis and bone development.^3^, ^4^ In addition, IL‐15 contributes to the development of muscle, promotes muscle damage repair and myofiber synthesis,^5^, ^6^, ^7^, ^8^, ^9^ promotes skeletal muscle mitochondrial biogenesis, and enhances skeletal muscle energy supply (Figure 1).^10^, ^11^, ^12^ Previous studies have focused on exogenous IL‐15‐induced immune factors in vivo and the regulation of cellular biological functions by IL‐15 ex vivo (Figure 2); however, few studies have focused on the in vitro environment.^13^, ^14^ There is evidence that different exercise patterns affect the synthesis and secretion of IL‐15 in the blood and skeletal muscle.^15^, ^16^, ^17^ However, few studies have evaluated changes in the function of skeletal muscle, bone, and other organs with variation in IL‐15 levels. Furthermore, despite the well‐established effect of exercise on bone and skeletal muscle, it is not clear whether these effects are mediated by IL‐15. Accordingly, we considered the regulatory relationships among exercise, IL‐15, and related biological functions. In this review, we explore the trends in IL‐15 expression under different exercise modes, its effects on bone and skeletal muscle, and, ultimately, its role in human motor function.

## 2 STRUCTURE AND RECEPTORS OF IL‐15

Grabstein et al.^1^ isolated IL‐15, a cytokine with a molecular mass of 14–15 kDa, from the supernatant of the monkey kidney epithelial cell line CV‐1/EBNA. IL‐15 consists of a precursor protein of 162 amino acids, of which 48 amino acids make up the leading sequence and the 114‐amino‐acid mature IL‐15 subunit includes two disulfide bonds and two N‐linked glycosylation sites.^18^ The amino acid homology of human and ape IL‐15 is 97%^1^ and that of human and mouse is 73%.^19^ The receptor for IL‐15 is a heterotrimer consisting of three subunits, IL‐15 receptor‐α (IL‐15Rα; CD215), IL‐2Rβ/IL‐15Rβ (CD122), and IL‐2Rγ (CD132).^20^ IL‐15 binds to its receptor via the *α* subunit to activate the other two subunits (*β* and *γ*).^20^ Since IL‐15 shares the *β* and *γ* chains of IL‐2R,^21^ IL‐15 and IL‐2 may share similar functions and characteristics.^1^, ^22^

## 3 IL‐15 SYNTHESIS AND SECRETION

IL‐15 is produced by many types of cells, including macrophages, epithelial cells, fibroblasts and skeletal muscle cells.^21^ As evaluated by nucleic acid hybridization of human tissues, *IL‐15* mRNA expression levels are highest in the placenta and skeletal muscle.^1^ The most active form of IL‐15 in vivo was in a complex with IL‐15Rα.^23^ IL‐15Rα shows high affinity for IL‐15 and regulates the biological function and secretory activity of IL‐15.^24^

## 4 PHYSIOLOGICAL REGULATION OF IL‐15

Skeletal muscle, the largest metabolic organ of the body, secretes various cytokines.^25^ The secretion of these cytokines regulates metabolic activity within the muscle; additionally, via various pathways such as blood circulation, cytokines influence other tissues and organs. Studies have shown that IL‐15 is highly expressed in skeletal muscles, can be secreted by skeletal muscle, and can mediate the physiological activity of the bones.^26^ The skeletal muscle and bone are important components of the locomotor system, suggesting a significant link between IL‐15 and the locomotor system. Recent studies have shown that IL‐15 is closely associated with metabolic processes in the bone and skeletal muscle. In addition, as an indispensable member of the interleukin family, IL‐15 plays a pivotal role in the complex network of the immune system. It not only plays a central regulatory role in key biological processes such as proliferation, differentiation and activation of immune cells, but is also crucial for the maintenance of homeostasis and normal functioning of the immune system.

### 4.1 Effects on bone metabolism

There are strong associations between single nucleotide polymorphisms in human *IL‐15* and *IL‐15Rα* and bone density and bone mass.^27^ IL‐15Rα knockout (*IL‐15Rα* ^*−/−*^) mice have significantly reduced osteoclastogenesis, a significant increase in femur and spine bone density, and an improvement in bone microarchitecture.^28^ Ovariectomized female*IL‐15Rα* ^*−/−*^mice show a significantly higher bone mineral density than that of wild‐type (WT) mice and an altered bone microarchitecture, suggesting that IL‐15Rα plays a role in preventing osteoporosis.^28^ However, conflicting results regarding the effects of IL‐15Rα have been obtained. Micro‐CT analyses of the femurs of *IL‐15Rα* ^*−/−*^ mice have shown that bone mineralization is impaired and the bone volume fraction, number of bone trabeculae, bone thickness, osteoblast differentiation capacity and density of osteocytes in the cortical bone of mice are significantly lower than those of control mice.^3^ Transcriptomics and bioinformatics analyses have revealed that IL‐15Rα is also closely linked to bone mineralization, suggesting that IL‐15/IL‐15Rα signalling is essential for bone mineralization and phosphate homeostasis.^3^ The discrepancy among studies may be explained by the impaired osteoclast activation signalling in the former experiment, differences in the age and sex of mice between studies (given sexual dimorphism in the skeletal system), and differences in detection methods.

Quinn et al.^4^ constructed the HSA‐IL2SP‐IL15 line of transgenic mice with a high secretion efficiency by replacing the inefficient IL‐15 long signal peptide with a more efficient IL‐2 signalling sequence and by eliminating several post‐transcriptional check sites that block the efficient translation of IL‐15.^29^ HSA‐IL2SP‐IL15 mice overexpressed skeletal muscle IL‐15 at the mRNA and protein levels and had significantly higher serum IL‐15 levels and bone mineral contents than those of WT mice. Again, this suggests that IL‐15 increases bone inorganic components and strengthens bone stiffness. Mouse monocyte macrophage leukaemia cells were stimulated using IL‐15 and/or receptor activator of nuclear factor‐κB ligand (RANKL), revealing that RANKL‐induced osteoclast formation and bone resorption were significantly higher after the addition of IL‐15 than after treatment with RANKL alone.^30^ These results suggest that the two factors synergistically promote osteoclastogenesis and that IL‐15 may have a positive effect by activating the ERK signalling pathway.^30^ Other studies have also shown that IL‐15 promotes osteoclastogenesis. Conflicting results regarding the effects of IL‐15 on bone may be explained by unintended effects of genetic alterations on biological traits. Furthermore, the in vitro injection of IL‐15 does not provide insight into survival. IL‐15 may exert a bidirectional regulatory effect according to the physiological or pathological state, with distinct effects on the proliferation and differentiation of osteoblasts or osteoclasts and the deposition of skeletal matrix or resorption of damaged bone tissue. In this way, IL‐15 can dynamically alter metabolic processes in the human skeleton and ultimately mediate the function of the locomotor system.

### 4.2 Effects on skeletal muscle metabolism

IL‐15 is abundantly expressed and secreted by human skeletal muscle; it is involved in muscle growth and synthesis.^31^ IL‐15 reduces protein degradation in muscle^5^ and regulates the interaction between muscle and adipose tissue.^6^ Kang et al.^7^ found that IL‐15 inhibits intramuscular fat infiltration and affects the differentiation of fibro‐adipogenic progenitors (FAPs). FAPs can differentiate into fibroblasts or adipocytes. They cause muscle fibrosis or fat infiltration, resulting in fibrosis and steatosis after acute and chronic muscle injury.^32^, ^33^ Adipocyte deposition in muscle fibres, resulting in fat infiltration, is irreversible and impairs muscle elasticity and thus muscle mass and function.^34^, ^35^ Fibrosis is considered a hallmark of the repair process after muscle damage.^36^, ^37^ FAPs exert bidirectional effects after muscle injury. Although they lead to fibrosis or fat infiltration in injured muscles, FAP activation is important for the differentiation of myogenic progenitors (MPs) and muscle regeneration.^38^ IL‐15 stimulates FAP proliferation via the Janus kinase (JAK)‐signal transducer and activator of transcription (STAT) pathway, promoting collagen deposition in myofibers and directly inhibiting FAP lipogenic differentiation in vivo and in vitro, which ultimately promotes myofiber regeneration.^7^ Therefore, IL‐15 can regulate the direction of FAP differentiation, providing a potential therapeutic strategy for chronic muscle injury.^7^

Figueras et al.^8^ found that the ratio of gene expression levels of pro‐apoptotic *Bax* to anti‐apoptotic *Bcl‐2* is elevated in skeletal muscle of rats with hepatocellular carcinoma and that IL‐15 administration attenuates this increase, suggesting that IL‐15 inhibits tumour‐mediated apoptosis in skeletal muscle fibres.^8^ Quinn et al.^39^ reported that insulin‐like growth factor‐I (IGF‐I) influences skeletal muscle metabolism via the proliferation and differentiation of myoblasts. This is quite different from the metabolic regulation of skeletal muscle by IL‐15, which induces the differentiation and accumulation of myosin heavy chain and α‐actinin in myotubes, promotes myofibril protein synthesis, inhibits tumour‐mediated apoptosis in skeletal muscle, and inhibits protein catabolism, thus contributing to the maintenance of myofiber growth and development.^9^ These findings suggest that IL‐15 can promote muscle damage repair as well as myofiber regeneration by regulating the differentiation capacity of FAPs. It also inhibits myofiber apoptosis and induces the differentiation and accumulation of myosin heavy chain and α‐actinin, which in turn maintains myofiber growth.

Peroxisome proliferator‐activated receptor δ (PPAR δ), a nuclear hormone receptor, is highly expressed in skeletal muscle and promotes myofiber type transition, increased skeletal muscle mitochondrial biogenesis, and energy metabolism.^40^ Proliferator‐activated receptor γ co‐stimulatory factor 1α (PGC‐1α) can regulate mitochondrial biogenesis by binding to and activating mitochondrial transcription factor A (TFAM), and nuclear respiratory factor‐1/2 (NRF‐1/2).^41^ Both PPAR δ and PGC‐1α are essential for energy metabolism and mitochondrial function in skeletal muscle. Studies have found that PPAR δ and PGC‐1α protein expression are elevated in skeletal muscles of mice with muscle‐specific IL‐15 overexpression.^4^, ^42^ Furthermore, a significant increase in the expression of PPAR δ was detected in IL‐15 knockout mice after the intraperitoneal injection of exogenous IL‐15.^43^ In C2C12 myoblasts, when IL‐15 was added at a dose of 100 ng/mL for six consecutive days, the mRNA expression levels of *PPAR δ* and *PGC‐1α* were significantly increased.^44^ These results suggest that IL‐15 enhances skeletal muscle mitochondrial biogenesis by promoting the expression of PPAR δ and PGC‐1α, which in turn increases the skeletal muscle energy supply.

### 4.3 Effects on immunity

IL‐15 plays a central role in immune system regulation, including the innate and adaptive immune systems.^45^ The innate immune system is the first line of defence and includes natural killer (NK) cells, dendritic cells and neutrophils, which respond rapidly and destroy pathogens. The adaptive immune system includes T and B cells, which have memory functions and provide long‐term immune protection.

IL‐15 promotes NK cell activation and proliferation via JAK1/3, STAT1/3/5, and protein kinase B (AKT)‐spliced X‐box binding protein 1 (XBP1) signalling pathways.^46^, ^47^, ^48^ IL‐15 also increases NK cell production of interferon (IFN)‐γ, which improves their killing activity and enhances the immune response.^49^ Budagian et al.^50^ found that IL‐15 promotes dendritic cell expression of co‐stimulatory molecules and IFN‐γ, optimizing their activation of NK and CD8+ T cells and thereby improving immune responses. In addition, IL‐15 induces morphological changes in neutrophils, enhances phagocytosis and anti‐microbial responses, and can effectively delay neutrophil apoptosis via anti‐apoptotic and kinase signalling pathways.^51^ IL‐15 stimulates neutrophil IL‐8 production and secretion through the activation of nuclear factor‐κB (NF‐κB), leading to the additional recruitment of neutrophils to sites of inflammation and amplification of the inflammatory response, mediating immune processes.^51^, ^52^

Notably, IL‐15 has a chemotactic effect on T cells; it can promote the activation and proliferation of T cells and enhance the activity of cytotoxic T lymphocytes, thus strengthening the ability to resist viral infection.^53^ In addition, IL‐15 can induce the differentiation of CD4+ T cells, promote their transformation to helper T cells, and promote the secretion of cytokines, such as IFN‐γ.^54^ For CD8+ T cells, IL‐15 not only enhances the killing activity of CD8+ T cells, but also maintains the survival and homeostatic proliferation of memory CD8+ T cells, providing long‐term protection.^55^, ^56^ As a pleiotropic cytokine,^57^ IL‐15 has the ability to induce the proliferation of B cells and promote their synthesis of immunoglobulin, thereby strengthening immunity.^58^

## 5 RELATIONSHIP BETWEEN IL‐15 AND EXERCISE

IL‐15 can be synthesized and secreted by skeletal muscles, and its content changes after muscle contraction. In addition, IL‐15 is extensively involved in anabolic processes in skeletal muscles.^59^ Therefore, IL‐15 is considered to be a myokine.^60^ Skeletal muscle is an active force‐generating organ of the locomotor system and affects locomotion‐related organs through muscle contraction. Its mass and function are also significantly regulated by exercise, suggesting that there is a complex link between human exercise and IL‐15 synthesis. Because of the diversity of forms and types of exercise, the effects of exercise on the human body are varied and complex. Thus, a general classification system according to commonly used exercise regimens as well as the intensity and loading characteristics of each exercise is adopted to discuss the effects of IL‐15, together with the results of a meta‐analysis. A description of the types of exercise and the corresponding changes in IL‐15 levels is presented in Table 1.

**TABLE 1:** Relationship between IL‐15 and exercise.

Reference	Sample type	Subject characteristics	Exercise form	Exercise intensity	Detection timing	Changes in IL‐15
Nielsen et al.^59^	Vastus lateralis, plasma	Males, in good health, regular physical activity (*n* = 8)	Resistance exercise	Leg press and knee extension (4 sets, 6–14 reps, 90 s rest between sets, 3 min rest between both movements)	24 h after exercise	Significant increase in muscle *IL‐15* mRNA, no significant change in plasma IL‐15 concentration
Pérez‐López et al.^83^	Vastus lateralis	Males, resistance trained (*n* = 14)	Resistance exercise	Four sets of 8–15 reps, 75% 1RM reps of bilateral leg push‐ups and knee extensions	24 h after exercise	Muscle *IL‐15* mRNA increased significantly
Nieman et al.^84^	Plasma, vastus lateralis	Males, strength athletes (*n* = 15)	Resistance exercise	Single 40% 1RM × 10 and 3 reps 60% 1RM × 10 with 2–3 min of rest (bench press, incline bench press, etc.)	2 h after exercise	Muscle *IL‐15* mRNA and plasma IL‐15 levels did not change significantly
Louis et al.^85^	Vastus lateralis	Males (*n* = 4), females (*n* = 2), nonsmoking, nonobese, and physically active volunteers	Resistance exercise	Three sets, 70% 1RM, 10 reps per set, bilateral knee extensions	Pre‐exercise, immediate post‐exercise, and 1, 2, 4, 8, 12, and 24 h post‐exercise	Muscle *IL‐15* mRNA did not change significantly
Bazgir et al.^86^	Serum	Males, non‐athlete, young (*n* = 14)	Resistance exercise	Three sets, 8–10 reps per set, 60 s rest between sets (deep squats, chest presses, etc., 70%–100% 1RM). Repeat after a 4‐day interval	Before and immediately (1–5 min) following exercise	IL‐15 increased significantly
Knuiman et al.^87^	Serum	Healthy males (*n* = 13)	Resistance exercise	2 days (5 sets × 8 reps, 80% 1RM), repeated bilateral leg push‐ups and leg extensions with 2 min of rest between sets	1 h after exercise	IL‐15 increased significantly
Riechman et al.^88^	Plasma	Males (*n* = 76), females (*n* = 77)	Resistance exercise	10 weeks, 3 times a week (80% 1RM, chest press, seated row, etc., 3 sets × 6–10 reps)	Immediately after (within 5 min) the first and last exercise session	IL‐15 increased significantly
Louis, et al.^85^	Gastrocnemius muscle	Males (*n* = 5), females (*n* = 1), ran 3–5 times per week	Aerobic exercise	Treadmill running (30 min, 75% VO_2max_)	8 h after exercise	Muscle *IL‐15* mRNA increased significantly
da Silva Soares et al.^89^	Vastus lateralis	Males (*n* = 7), females (*n* = 6), type 2 diabetes mellitus non‐insulin dependent	Aerobic exercise	12 weeks, 3×/week (40 min, moderate to high intensity running), 500 kcal reduction in daily energy intake	Start of exercise and 12 weeks after exercise	Muscle *IL‐15* mRNA increased significantly
Garneau et al.^90^	Plasma	Obese women (BMI ≥30 kg/m^2^), healthy women (BMI 22–29.9 kg/m^2^)	Aerobic exercise	Cycling (60 min, 60% VO_2_peak)	Every hour after exercise until 24 h.	IL‐15 increased significantly
Bartlett and Duggal^91^	Serum	Sedentary older adults (*n* = 25), physically active older adults (*n* = 25)	Aerobic exercise	Sedentary group (walking 2500–4500 steps per day), active group (walking 10,500–15,000 steps per day)	After walking	Serum *IL‐15* levels were significantly higher in the active group than in the sedentary group
Yargic et al.^92^	Serum	Professional athletes (*n* = 26)	Aerobic exercise	35 km track + climbing 940 m	Day before and within 15 min after the race	IL‐15 increased significantly
de Sousa et al.^93^	Plasma	Amateur marathoners (*n* = 40–43, age 41.1 ± 0.9 years, weight 74.8 ± 2.7 kg)	Aerobic exercise	One full marathon	Immediately and 72 h after exercise	IL‐15 decreased significantly
Pérez‐López et al.^94^	Serum	Menopausal obese women (*n* = 10, 50–65 years, BMI >25 kg/m^2^)	Aerobic exercise	12 weeks × 3 sessions × 60 min (55–75% heart rate reserve, treadmill, elliptical, and rebounder exercise)	Before and after the 12‐week intervention	IL‐15 decreased significantly
Kim et al.^75^	Serum	Males with advanced prostate cancer (*n* = 9, age 67.8 ± 10.1 years)	High‐intensity interval exercise	Power Pedal × 2 sets (6 times × 4 min, 70–85% HRmax +5 times × 2 min, 50%–65% HRmax +5 min cooldown)	Immediately and 30 min after exercise	Serum IL‐15 protein elevated immediately after exercise and returned to baseline after 30 min
He et al.^76^	Serum	Healthy non‐athletic men (23 ± 3 years) (*n* = 17)	High‐intensity interval exercise	Two sets, 6 reps, 30 s, (running bouts at 100% Vmax, with 90 s of active recovery between bouts)	Baseline and 0, 1, 3, 24, 48, and 72 h after exercise	No significant change
Coletta et al.^77^	Plasma	Menopausal women with overweight or obese status (*n* = 33, BMI ≥25 kg/m^2^)	High‐intensity interval exercise	12 weeks HIIT (4 × 4 min, 90–100% HR peak +3 × 3 min, 50%–70% HR peak), MICT (41 min, 60–70% HR peak)	Baseline and end‐of‐study	No significant difference
Molanouri Shamsi et al.^78^	Gastrocnemius muscle	Tumour‐bearing mice (*n* = 8, 6–8 weeks old)	High‐intensity interval exercise	6 weeks × 5 days/week (10 reps, 2 min 70% *V*_max_ +9 reps, 2 min 50% *V*_max_)	24 h after the last workout	Muscle IL‐15 protein increased significantly
Han et al.^79^	Soleus muscle	Exercise group (18–26‐month‐old female rats, *n* = 6); control group (26‐month‐old female rats, *n* = 12)	High‐intensity interval exercise	8 months, 5 days/week (4 × 15 m/min + 1 × 25 m/min, alternating 9 times)	48 h after the last workout	*IL‐15* mRNA increased significantly
Nishida et al.^80^	Serum	Postmenopausal women (*n* = 62, 85–31 years)	Other exercise training methods	Step exercise, 12 weeks, 3 times/day (10–20 min, 15–20 cm steps, 40 steps/min, step rate increasing by 10 steps/min every 4 min, with 2‐min rest intervals)	Before and after the 12‐week intervention	No significant difference
Roh et al.^81^	Plasma	Overweight and obese adolescents (*n* = 20, age 12.55 ± 0.51 years, BMI 24.33 ± 1.74 kg/m^2^)	Other exercise training methods	Taekwondo and other basic movements, 16 weeks, 5 times/week× (60 min)	Before and after the 16‐week intervention	No significant difference
Iki et al.^82^	Saliva	Patients with cerebrovascular disease, requiring substantial help with daily movements, assisted feeding, and less than 14 h per week out of bed (*n* = 21)	Other exercise training methods	Active‐assisted exercise (10 min each in prone and seated positions, 100 flexions and extensions of the limbs)	Before the intervention (0 h) and 0.5, 1, and 3 h after the end of the intervention	No significant difference

### 5.1 Resistance and aerobic exercise training

Resistance exercise (RE) is a training method that improves muscle tone by increasing the resistance of the muscle during contraction. This type of training promotes an increase in the cross‐sectional area of the muscle, thus enhancing the muscle's ability to contract.^61^, ^62^ As an exercise method used on a daily basis, it is important in improving muscle mass, enhancing bone stiffness, and improving the function of the locomotor system.^63^, ^64^ Aerobic exercise (AE), a training method aimed at improving the body's ability to work for long periods of time, enhances cardiorespiratory fitness, promotes angiogenesis, regulates mitochondrial function and improves the muscle oxidative capacity.^65^ There is a close link between this form of exercise and IL‐15 expression. In recent years, there have been numerous extensive and in‐depth studies on the interaction between RE, AE and IL‐15. However, the conclusions of these studies have not yet formed a unified consensus. To deeply analyse and distill more professional and widely referenced conclusions, this section incorporates all meta‐analysis literature on the relationship between IL‐15 and exercise for in‐depth discussion.^66^, ^67^, ^68^, ^69^

The meta‐analysis by Bettariga et al.^66^ investigated participants that performed single bouts of exercise according to different exercise types (RE, AE or combined exercise). Although there was no significant change in blood IL‐15 concentrations in a healthy population following RE, a single bout of exercise increased blood IL‐15 concentrations in the period immediately to 24 h following exercise. However, this change was not significant and may be attributed to the large variation in effect sizes.^66^ In a meta‐analysis published in 2024, researchers found a slight‐to‐moderate effect of acute RE on IL‐15 levels,^67^ demonstrating a small positive average effect for IL‐15. The study by Khalafi et al.^69^ noted that compared to baseline, acute RE and AE (exercise duration ranged from 3 to 360 min, while the intensity was widely different for each mode of exercise) increased circulating IL‐15 concentrations immediately after exercise. Acute exercise was associated with an increase in IL‐15 concentration even 1 h after exercise.^69^

In a subgroup study of the effect of chronic exercise on IL‐15 levels, Khalafi et al.^69^ noted that prolonged exercise (exercise duration≥2 weeks) did not have a significant effect on IL‐15 concentrations and was independent of the type of exercise. Of note, Prado et al.^68^ examined chronic exercise (AE, RE or combined exercise) and showed for exercise intervention characterization that intervention durations of <16 weeks decreased IL‐15 levels, whereas intervention durations of ≥16 weeks had no effect on IL‐15 levels. Analysis of individual characteristics showed that individuals aged ≥65 years had reduced blood IL‐15 concentrations after the exercise intervention. Short‐term exercise interventions, particularly with an older intervention group, may not have been adapted or been poorly tolerated by the body during the exercise period, and the increase in blood volume and accelerated blood flow due to exercise may have promoted further diffusion and dilution of IL‐15, potentially leading to a decrease in the detected circulating IL‐15 concentration. In addition, this meta‐analysis also centered on the effects of physical activity, age, and BMI on IL‐15 concentrations.^68^ For physical activity, populations (physically active, non‐physically active) were divided according to regular physical activity performed for at least 6 months, or had a proven specific intensity or volume of exercise in each bout or in each week. This cross‐sectional study found no significant differences between circulating concentrations of IL‐15 in physically active and non‐physically active adults. This may be because only a small number of total studies were included, the majority of subjects were middle‐aged or older and exhibited a lower exercise intensity, and the total duration of none of the training periods was reported. These may have contributed to the fact that physical activity had no significant effect on the population. In terms of age profile, older adults had lower circulating IL‐15 levels than young and middle‐aged adults, a phenomenon that seems to be reflected only in normal‐weight populations (being <25 kg/m^2^ normal weight, 25–30 kg/m^2^ overweight and > 30 kg/m^2^ obese). In addition, upon dividing the population according to BMI (normal, overweight and obese), further analysis revealed that circulating IL‐15 levels were significantly higher in overweight older adults than in overweight young and middle‐aged adults. The changes in IL‐15 concentrations in overweight older individuals may be related to the body's compensatory mechanisms. Abnormal functional states of the body may lead to damaged receptors or target cells of IL‐15, which cannot receive relevant signals and subsequently exacerbate IL‐15 secretion. Overall, this meta‐analysis concluded that IL‐15 concentration showed a decreasing trend in the subgroups of short‐term exercise intervention and older intervention.^68^ This suggests that the duration of the exercise intervention and the age of the subjects may be important factors influencing IL‐15 concentrations.

In the subgroup analyses on RE and AE, the focus of each meta‐analysis, time of publication, and studies that housed them varied, resulting in some differences in data characteristics such as subjects, interventions, exercise characteristics and testing time. However, it still provides a valuable reference point for us to study the effects of two types of exercise on IL‐15 concentration. According to the data disclosed in the four meta‐analyses, the quality scores of the included studies were low and the internal heterogeneity of the articles was high. This may be related to the large variations in the exercise protocols of the included studies as well as the significant differences in the subjects' exercise intervention cycles. Therefore, it is crucial to ensure the uniformity of exercise regimen and intensity in subsequent studies on the regulation of IL‐15 secretion by exercise. In view of the short half‐life of IL‐15, it is crucial to accurately determine its detection time to analyse the relationship between exercise and IL‐15 levels. Notably, as an important myokine secreted by skeletal muscle, the expression level of IL‐15 in muscle can directly and significantly reflect the dynamic changes after exercise.^60^ However, the practical limitations of sampling IL‐15 in skeletal muscle and the potential damage to the human body have somewhat constrained the development of large‐scale studies. In view of this, future research should be devoted to exploring more in‐depth and intuitive scientific methods to monitor the expression of IL‐15 in the body, while at the same time trying to mitigate or avoid the damage caused to the subjects and finding effective compensatory strategies to promote the further development of this field of research.

Although the conclusions drawn from the above studies are not very consistent, there is some reference value in terms of the role of acute exercise in promoting IL‐15 secretion. In particular, acute RE showed a more significant tendency to promote IL‐15 secretion. In contrast, the role of chronic RE and aerobic exercise on IL‐15 content is uncertain. Combined with the previously discussed regulatory effects of IL‐15 on bone and skeletal muscle, it is hypothesized that IL‐15 may mediate the effects of exercise and training on physiological parameters and organismal functions. RE may play a positive role in the locomotor system by regulating skeletal muscle or circulating IL‐15 concentrations, which in turn affects metabolic processes in bone and skeletal muscle.

### 5.2 High‐intensity interval exercise training

In recent years, enthusiasm for simple and easy exercise activities that can be performed at any time without the need for specialized equipment and strict environmental requirements has shifted the focus to high‐intensity interval training (HIIT). HIIT is characterized by a set of short or long exercise periods (30 s to 4 min) at high intensity (>85% VO_2max_), followed by a recovery period (30 s to 4 min).^70^ This approach improves vascular function, accelerates blood circulation,^71^ promotes mitochondrial function,^72^ regulates glucose and lipid metabolism,^73^ and improves insulin sensitivity.^74^

In a study of patients with prostate cancer assigned to two groups of HIIT (6 times × 4 min, 70%–85% HR_max_ +5 times × 2 min, 50%–65% HRmax +5 min cool down, *n* = 9), the IL‐15 content in the blood was significantly elevated in the immediate post‐exercise period compared to baseline and gradually recovered to baseline levels after 30 min of rest.^75^ Alternatively, a study of healthy men did not reveal significant changes in blood IL‐15 levels after HIIT (2 × 6 sets, 30 s, 100% *V*_max_, *n* = 17).^76^ Coletta et al.^77^ compared 12 weeks of HIIT (4 × 4 min, 90%–100% HR peak +3 × 3 min, 50%–70% HR peak, *n* = 33) and moderate‐intensity continuous training (41 min, 60%–70% HR peak, *n* = 33) in postmenopausal women with overweight or obese status, revealing no significant difference in circulating levels of myokines between the two types of exercise and slightly lower or similar plasma IL‐15 levels to those in controls.

However, there are discrepancies in the effect of HIIT on IL‐15 in animal experiments. In a study of tumour‐bearing mice that were randomly divided into control and HIIT (5 × 10 repetitions, 2 min, 70% *V*_max_ +5 × 9 repetitions, 2 min, 50% *V*_max_, *n* = 8) groups, IL‐15 protein expression was increased in the gastrocnemius muscle of HIIT mice after 6 weeks of exercise.^78^ Han et al.^79^ observed that sustained HIIT (4 × 15 m/min + 1 × 25 m/min, nine alternating repetitions, *n* = 18) in rats for 8 months (5 days per week) resulted in a significant elevation of *IL‐15* mRNA in the flounder muscle compared with levels in controls. Due to the specificity of HIIT, relatively few experiments have focused on humans. In addition, the study subjects, method of IL‐15 detection, and expression site in animal experiments differ between animal and human studies; accordingly, the reference value of these studies is limited. At present, definitive results about the trend in IL‐15 after HIIT are lacking, and further studies are needed to elucidate the effect of HIIT on IL‐15.

### 5.3 Other exercise training methods

Various less common exercise types have been evaluated. Nishida et al.^80^ reported that 12 weeks of home step exercise (3 times/day × 10–20 min, step height 15–20 cm, *n* = 62) on myokinesis in postmenopausal women does not affect serum IL‐15 levels. In a study of overweight adolescents, individuals who underwent taekwondo training (16 weeks × 5 sessions/week × 60 min, *n* = 20), a popular physical activity among adolescents, showed no differences in IL‐15 levels from those in controls.^81^ Few studies have evaluated movement patterns in individuals with limb dysfunction. Among these studies, Iki et al.^82^ evaluated individuals who were bedridden, mainly due to cerebrovascular disease, revealing that when patients bent and stretched their arms and legs as much as possible in the sitting and supine positions, vital signs improved significantly and IL‐15 levels in the saliva increased. Although the increase in the salivary IL‐15 levels was not significant, these results suggest that IL‐15 levels may change after exercise.

Taken together, studies of nontraditional exercise modalities show significant differences in terms of exercise content, detection methods and participant characteristics. These differences likely contribute to inconsistencies in observed effects on IL‐15 expression in vivo. In addition, the secretion mechanisms, release processes, and regulatory centers of IL‐15 during such exercises are not well understood, requiring further in‐depth studies. Future studies should adopt a more detailed and systematic approach to unravel the mechanisms by which IL‐15 contributes to exercise physiology.

## 6 CONCLUSION AND OUTLOOK

IL‐15 is a key factor in the prevention and treatment of muscle atrophy and degeneration as well as osteoporosis, infection, and other chronic diseases; however, upstream regulators, downstream effectors and detailed signalling pathways have not been comprehensively characterized. The effects of IL‐15 and its receptor on mitochondrial dynamics in skeletal muscle are unclear, and multi‐omics analyses are a promising approach to address this gap in knowledge. Additionally, further studies are needed to investigate the effects of IL‐15 on skeletal metabolic homeostasis in different states.

In conclusion, the effects of exercise on physical function may be mediated by IL‐15. In particular, IL‐15 is mainly expressed in the locomotor system and contributes to various biological processes under different exercise modalities. Exercise continuously regulates the dynamic balance of the skeletal and skeletal muscle microenvironment through IL‐15 to maintain the different states of these organs. In addition, there is a close link between the maintenance of human immune function and exercise‐mediated IL‐15 expression. However, the mechanisms underlying the role of IL‐15 in motor system regulation remain unclear. Future investigations on the relevant links between IL‐15 and motor endplate‐mediated neuromodulation, which is closely related to exercise, are needed to elucidate the specific neural pathways and target‐organ effects of the central nervous system in controlling IL‐15 secretion, providing novel targets for improving human health. Little is known about the connection between musculoskeletal crosstalk and locomotion, which is closely related to IL‐15, and this may be an important direction for future research.

## AUTHOR CONTRIBUTIONS

**Ziqiang Duan:** Writing – original draft (equal); writing – review and editing (equal). **Yang Yang:** Writing – review and editing (equal). **Mianhong Qin:** Validation (equal); visualization (equal). **Xuejie Yi:** Funding acquisition (equal); methodology (equal); project administration (equal); supervision (equal); visualization (equal); writing – review and editing (equal).

## FUNDING INFORMATION

This work was supported by the National Natural Science Foundation of China (No. 12072202).

## CONFLICT OF INTEREST STATEMENT

The authors confirm that there are no conflicts of interest.

## Data Availability

Data sharing not applicable—no new data generated, or the article describes entirely theoretical research.
